# Global Longitudinal Strain Monitoring to Guide Cardioprotective Medications During Anthracycline Treatment

**DOI:** 10.1007/s11912-022-01242-y

**Published:** 2022-03-03

**Authors:** Thomas H. Marwick

**Affiliations:** 1 Baker Heart and Diabetes Institute, PO Box 6492, Melbourne, Victoria 3004 Australia; 2 Baker Department of Cardiometabolic Health, University of Melbourne, Melbourne, Victoria Australia

**Keywords:** Global longitudinal strain, Cardioprotective medications, Anthracycline treatment

## Abstract

**Purpose of the Review:**

Anthracycline chemotherapy carries a risk of myocardial dysfunction and heart failure even at relatively low doses, and the clinical prediction of cancer treatment-related cardiac dysfunction (CTRCD) is inexact. Careful imaging or biomarker surveillance during chemotherapy can identify CTRCD before the development of heart failure. Currently, this surveillance is performed using ejection fraction (EF). While this is a reliable and reproducible test with three-dimensional techniques, the most widely used imaging technique is two-dimensional echocardiography, for which EF measurements have broad confidence intervals.

**Recent Findings:**

The use of global myocardial strain (GLS) provides a more reliable and reproducible means of assessing global cardiac function and shows meaningful changes before a significant change of EF. Observational studies have shown that although absolute measurements of GLS, both at baseline and during therapy, are predictive of CTRCD risk, the most reliable approach is to assess the change of GLS with therapy — a meaningful relative change of 10–15% being significant. A clinical trial comparing GLS to EF surveillance did not show a significant change of EF in the overall study group, but did show that patients managed with a the GLS-guided approach were less likely to develop a meaningful change of cardiac function to an abnormal level. In at-risk patients, there is good evidence for the protective value of neurohormonal antagonists and statins: the use of GLS enables these benefits to be directed to those most likely to benefit, while minimizing their use in the majority of people, who do not need them.

**Summary:**

Although GLS requires an element of training and efforts to ensure uniformity, it has proven to be a feasible, robust, and reproducible technique, ready for wide adoption.

## Introduction

The increasing survival from cancer is leading to a huge cohort of cancer survivors [1]. Among these individuals, the development of heart failure (HF) during follow-up is a significant source of morbidity. The prognostic implications of HF due to chemotherapy seem to be worse than most other types of HF [2]. Prevention and early recognition may avoid the scenario of the successfully treated cancer patient succumbing from HF.

## Defining Cancer Treatment-Related Cardiac Dysfunction (CTRCD)

The condition that we are seeking to avoid is the development of HF in cancer survivors. While this may occur acutely, it is more commonly detected years after chemotherapy. Of course, awaiting the clinical presentation of HF is not a prudent approach to this diagnosis, because it takes so long to develop and because the patients at that stage have disease that is too far advanced make a therapeutic impact. Consequently, the definition of CTRCD is based upon a meaningful drop of ejection fraction (10% if asymptomatic, 5% if symptomatic) to below the normal range, albeit with some differences in exact definitions [3].

The cause of LV dysfunction in cancer patients is inherently multifactorial, with significant contributions from not only chemotherapy, but also risk factors that are shared between cancer and cardiac disease [4]. Nonetheless, patients treated with potentially cardiotoxic chemotherapy represent an important group for surveillance both acutely at the time of chemotherapy, as well as during long-term follow-up.

CTRCD may arise from exposure to cytotoxic agents or biological agents. While the classification into types I and II cardiotoxicity, respectively, has been criticized as being too simplistic, it has some value. CTRCD from biological agents is usually reversible, and uncommon — most studies suggest frequencies of 2% with lapatinib, imatinib, trametinib, and bevacizumab, although up to 11% is reported with sunitinib, and up to 27% with trastuzumab [5], possibly reflecting other influences. In contrast, myocardial injury due to cytotoxic agents, related to oxidative stress, free radical formation, and cell death, is associated with ultrastructural changes at biopsy, and is usually irreversible. CTRCD may occur at three stages in relation to chemotherapy — (i) acutely (a dose-dependent and usually reversible decline in LV function during/after infusion), (ii) subacutely (a rare, myocarditis-like presentation with edema, wall thickening, and diastolic dysfunction within a few weeks), and (iii) late (usually in 1st year but occurring in up to 10–20 years, with the dose-response influenced by age, sex, underlying CVD, hypertension, and smoking) [5]. As the development of HF or LV dysfunction is not purely a reflection of chemotherapy, but is also influenced by the background risk factor milieu, possible direct effects of cancer on the heart, and the impact of other treatments including radiotherapy, this needs to be approached on a probabilistic rather than a deterministic basis.

## Clinical Prediction

A Bayesian approach is necessary whenever imaging is used in risk assessment, and patients over the age of 65, those with previous anthracycline use and radiotherapy, those with coronary or other heart disease, and hypertension are particularly at risk of developing anthracycline-related cardiotoxicity [6]. The development of LV dysfunction and cardiotoxicity is dose-dependent — the frequency of HF attributable to anthracyclines, at least acutely, has decreased from the original report by Von Hoff [7]. Paradoxically, patients administered low doses of anthracycline account for the majority of cases, as although the rate of occurrence is low, these account for a very large number of patients, who are at some risk, even at doses < 250 mg/m^2^ [8]. The use and dose of anthracycline has been incorporated into various risk prediction tools, but although these do identify the spectrum of risk, they have been unable to reliably identify a very low risk group, who could forego imaging [9, 10].

The fundamental need for HF surveillance in this group is a solution that will work in large numbers of people (which has implications for cost and availability), at a high level of accuracy (taking note of the risks of misdiagnosis), at a high level of test-retest consistency, and irrespective of differing baseline risk. Three successive stages precede the development of HF — an initial phase where the causative pathway is identified, abnormal myocardial deformation, and asymptomatic LV dysfunction (Table 1). The use of more specific and less sensitive tests may detect disease at a late stage, whereas more sensitive and less specific tests have the potential of detecting early disease, but at the risk of mis-labeling patients as having disease.

**Table 1:** Identification of CTRCD at various clinical stages before the development of HF

Clinical phase		Imaging	Laboratory tests
Asymptomatic LVD		EF < 53%	BNP
Abnormal deformation		Abnormal LV strain	BNP
Causative factors	Myocyte injury	CMR	HsTn
	Oxidative stress		MPO
	Fibrosis		ST-2, galectin
	Inflammation		CRP, IL-6
	MicroRNA		miR-1, miR-29b, miR499

Legend: *BNP*, brain natriuretic peptide; *IL*, interleukin; *MPO*, myeloperoxidase

## Use of Ejection Fraction to Guide Therapy

Ejection fraction (EF) is part of the current definition of CTRCD, and is uniformly referenced in the guidelines [3, 11]. However, EF is not without its problems. EF is a useful marker of prognosis in patients with HF, particularly with an EF < 40%, but its association with outcome in individuals with preserved EF is rather poor. The diagnosis of CTRCD is based upon a meaningful *reduction* of EF to below the *normal range*, and there are challenges to both defining normal and defining reduction. The ability to interpret change can be confounded by alterations of loading conditions, implying that it may change despite the presence of stable myocardial function if BP changes in the course of serial follow-up. High and low heart rate, mitral regurgitation, and left bundle branch block all provide technical challenges.

Two-dimensional echocardiography is the most widely used technique for surveillance, and calculations of LV volumes using this technique are dependent on geometry, with changes from one visit to the next being influenced by different imaging planes. These relatively common differences in imaging planes lead to wide confidence intervals for the detection of sequential changes — absolute EF differences of up to 10% cannot be attributed to a change of myocardial status and may simply reflect measurement variation [12]. The definition of normal with different imaging modalities is different [13], and both sex and ethnic background influence findings [14].

EF may be calculated without geometric assumptions when a full 3-D data set is obtained, most commonly using 3D echocardiography or cardiac magnetic resonance. In contrast to the 10% confidence intervals of 2D echo, a meaningful change of 3D-EF is 5–6% [15]. However, although 3D-EF is recommended in guidelines, it is often not performed because of inadequate imaging windows and suboptimal image quality.

EF is widely assessed at baseline in patients undergoing anthracycline chemotherapy, with a recent data-linkage of chemotherapy for breast cancer with administrative data showing its use in > 85% at baseline. In contrast, only about 50% of patients have an echocardiogram performed during follow-up, and fewer than 50% have monitoring is recommended in guidelines [16, 17].

## Use of Strain to Guide Therapy

### Technical Considerations

Myocardial strain measures the magnitude of deformation of a defined length of myocardium during each cardiac cycle, referenced to the original length. Because the myocardium shortens, this is classically described as a negative number, but when strain is averaged in all myocardial segments (global longitudinal strain, GLS), it is always negative, and therefore can reasonably be expressed without a negative sign (recently coined “global longitudinal shortening”) [18]. This certainly facilitates communication with oncologists and other medical specialists, who may be unfamiliar with the derivation of this parameter.

The initial calculation of strain was performed using color tissue Doppler, which provided high temporal resolution, but had the disadvantage of being susceptible to noise, dependent on alignment with the Doppler beam, and being quite challenging to measure. However, the development of speckle strain, now two decades ago, has made the assessment of strain more feasible, reliable, and reproducible. Initial problems relating to inconsistencies of measured GLS by different machines, attributable to different methods of post-processing between manufacturers, have largely been addressed following a concordance process. The variability of strain between systems is now analogous to the variability of other common measurements such as LV dimensions [19•]. A number of studies have defined the normal range of strain in volunteers — a recent meta-analysis showed that a normal GLS is > 18% [20], but the interval between 16 and 18% should be considered borderline, in part because of the load-dependence of strain.

GLS has a well-defined learning curve, and is easy to learn, requiring a minimum of 50 patients to achieve expert competency (intraclass correlation coefficient > 0.9) in groups with varying levels of baseline skill over a period of 3 months [21]. Other studies have suggested an even shorter learning curve [22]. Prior background knowledge in echocardiography is an influential factor affecting the attainment of inter-observer reproducibility and time efficiency [21]. In contrast, short-axis strain analysis of global circumferential stain is more difficult and expert level was not achieved by the end of the study. Radial strain is inherently highly variable and probably should not be used outside of research settings.

In addition to the true error of the measurement, sources of variation may arise from patient characteristics (e.g., poor image quality due to body habitus and mastectomy is also a potential cause of underestimation), equipment (image acquisition and post-processing), and technical limitations on the part of the observer (particularly related to experience). The use of strain as a reliable physiologic marker is dependent on attention to detail. Imprecision in tracing the region of interest, inadequate tracking, failure to exclude the pericardium, and annulus are all potential source of variability and will lead to underestimation of GLS [23]. As GLS is load-dependent, some variation in sequential imaging may arise from differences in blood pressure — lower GLS measurements may be attributable to higher blood pressure measurements at the time of follow-up. The measurement of myocardial work accounts for loading conditions and may be a solution to this problem [24].

**Fig. 1:**
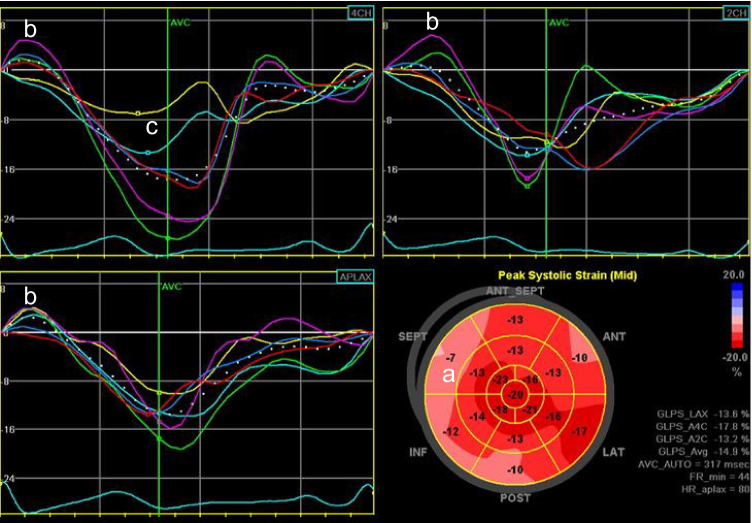
Underestimation of GLS due to technical difficulties. In this asymptomatic patient, biplane EF was 62% and GLS was reported to be −14%. Review of the polar map (**a**) shows heterogeneity of regional strain, disproportionately in the basal segments — this is an unusual pattern that should raise suspicion of artefact. Many of the strain curves (**b**) show an initial lengthening before shortening — this feature can be seen in the presence of scar, but again with be unusual in the setting of an apparently normal LV. Likewise, while heterogeneity of regional strain can be seen with diffuse processes, the magnitude of this variation is unusual. Revision of tracking demonstrated a normal GLS

The process of establishing strain in an echocardiography laboratory should start with a concordance process between the personnel doing the measurements. Although different laboratories may show minor but important differences in strain measurements [25], once conformity has been defined between observers, drift in strain measurements seems to be small [22]. Nonetheless, evaluation of tracking and the shape of the strain curves is an essential aspect of using GLS (Fig. 1), which should not be simply gathered from the polar map display.

### Application to Cardio-Oncology

A number of observational studies concerning the use of myocardial deformation have been reported over the last decade. The reliability of these for detecting CTRCD was reviewed in a recent meta-analysis [26••]. Impaired GLS both at baseline and during treatment have a high-sensitivity and modest but variable specificity for identifying CTRCD (Table 2). Because of the inter-individual variation of GLS, using each patient as their own control is a preferable approach. Absolute changes of GLS between 2–3%, and relative changes of between 10–15% have a sensitivity in the 80–90% range for the detection of CTRCD, with a specificity of 80%, albeit with some variability (Table 2).

**Table 2:** Sensitivity and specificity of GLS for predicting CTRCD. A, pre-treatment GLS measurements; B, Absolute on-treatment GLS measurements; C, Absolute difference in GLS; D, Relative change in GLS. Simplified from Oikonomou [26••]

	Author, date	Cancer type	Cutoff	Sensitivity	Specificity
Pre-treatment GLS	Charbonnel, 2017 Ali, 2016	Hematologic Hematologic	−20.0% −17.5%	83% 86%	72% 81%
Absolute on-treatment GLS	Milks, 2018 Sawaya, 2012 deAlmeida 2018 Charbonnel, 2017 Tang, 2016 Paraskevaidis, 2017 Guerra, 2016 Portugal, 2017 Negishi, 2013	Breast Breast Breast Hematologic Breast Hematologic Breast Breast Breast	−19.0% −19.0% −16.6% −17.5% −13.8% −18.4% −18.0% −18.0% −21.0%	64% 74% 80% 83% 86% 86% 89% 90% 96%	83% 73% 95% 72% 73% 71% 74% 45% 67%
Absolute diff in GLS	Fallah-Rad, 2011 Mornos, 2013 Charbonnel, 2017	Breast Mixed Hematologic	2.00% 2.77% 0.45%	80% 80% 83%	81% 73% 65%
Relative change in GLS (%)	Milks, 2018 Negishi, 2013 Sawaya, 2012 Charbonnel, 2017 Baratta, 2013 Florescu, 2014 Kang, 2013 Mornos, 2014 deAlmeida 2018	Breast Breast Breast Hematologic Mixed Breast Hematologic Mixed Breast	13.7% 11.0% 10.0% 2.26% 15.0% 9.0% 15.9% 13.7% 14.0%	45% 67% 78% 83% 86% 86% 86% 88% 100%	71% 95% 79% 65% 86% 81% 75% 71% 93%

Because of the tighter confidence intervals of GLS compared to EF, earlier changes of myocardial function may be detected with GLS. Very often, this means that GLS is a window to subsequent change of EF, effectively providing an opportunity for earlier, and thereby hopefully more effective intervention.

Nonetheless, it needs to be acknowledged that it is hard to prove prognostic benefit of one cardioprotective strategy, compared to others. In the SUCCOUR trial, 331 patients were randomized between GLS guided and EF guided cardiac surveillance [27•]. Both groups had an approximately 3% reduction of 3D-EF, which is within the reliability of the test. However, the development of cardiotoxicity was more common (13.7%) with EF guidance, compared with GLS guidance (5.8%, *p* = 0.02). This was primarily because 44 of 154 in the strain-guided group developed an abnormal response and were initiated on cardioprotective therapy, compared to 20 in the EF-guided group. The EF change in the 44 cardioprotection-treated, GLS guided patients was only 2.9%, compared to 9.1% in the EF-guided group, consistent with later (and therefore less effective) initiation of therapy.

## Alternative Surveillance Strategies

### Assessment of Diastolic Function

Diastolic dysfunction is a common manifestation of myocardial injury from various causes and can reasonably be expected to be a potential marker of CTRCD. Indeed, in a 2-year follow-up of 362 breast cancer patients, Upshaw et al. showed persistent worsening of diastolic function (manifest as reduced mitral annular velocities and reduced E/A ratio) [28]. Abnormal diastolic function was associated with a subsequent reduction of ejection fraction and worsening of GLS. This is a simple addition to the use of GLS and EF, but has the potential disadvantage that it is highly sensitive to other causes of myocardial injury, and therefore may be less specific than the other techniques.

### Biomarkers

Abnormal brain natriuretic peptide or high-sensitivity troponin have been reported with CTRCD and would certainly be more feasible than imaging for surveillance. Unfortunately, the levels of these biomarkers are also quite variable, implying that sampling has to be done at exactly the right time in order to identify myocardial injury. Moreover, not only cardiotoxicity but also other circulatory stress may lead to release of these biomarkers. Thus, while proposed as a potential cornerstone of surveillance [29], their reliability remains debated.

### Cardiac Magnetic Resonance (CMR)

The most reliable assessment of LV volumes and ejection fraction is obtainable with CMR, and this has been shown to be effective in predicting outcomes [30]. The variability of CMR-LVEF is similar to echocardiographic 3D-EF or GLS, and less than echocardiographic 2D-EF [31]. CMR-based GLS is feasible, but probably not superior to echo-based GLS. The role of CMR tissue characterization remains to be defined. A concerning aspect of these parameters is their variability [32], and although markers of fibrosis, inflammation and edema should be able to identify the earliest stages of myocardial injury; they may not be sufficiently specific for use in guiding treatment.

## Response to Abnormal Test

There are two potential responses to an abnormal test — initiation of cardioprotection, or modifying or interrupting cardiotoxic therapy. The latter is very much a last option because it may have a detrimental effect on treatment of the cancer, and is rarely required unless the patient has developed overt heart failure or other steps have failed.

Four groups of medications have been used for cardioprotection. Dextrazoxane has been known to have a cardioprotective effect during anthracycline chemotherapy for nearly 50 years, but its uptake has always been impeded by concerns that it may limit the efficacy of anthracyclines, and thereby inhibit successful cancer remission. While its activity was traditionally attributed to protection from oxidative stress (through iron chelation), it seems more likely that its efficacy pertains to differential expression and/or regulation of topoisomerase II isoforms in cardiac and cancer cells, leading to the selective modulation of anthracycline action [33]. Because this agent is thought to provide protection from injury, the model would be uniform use from time of initiation of chemotherapy, rather than selective use based upon abnormal imaging findings (see below).

In a systematic review of 25 studies with neurohormonal blockade using angiotensin-converting enzyme inhibitors, angiotensin receptor blockers, and beta-blockers, Elghazawy [34] showed similar levels of efficacy between the groups, with a 2.4% absolute difference in EF between treated and untreated groups. Their effectiveness for preserving EF was similar immediately after therapy, and at 6 and 12 months following treatment. These medications are presumed to work by unloading the LV, as they do in heart failure, as well as potentially through antioxidant and other metabolic effects.

The third group of agents that have been shown to be effective are statins, which are thought to limit the oxidative stress through which anthracyclines mediate cardiotoxicity. A recent meta-analysis of six studies [35] showed a 6% average difference in ejection fraction between treated and untreated groups, with a 60% odds reduction for developing cardiotoxicity. In a network meta-analysis, statins appeared to be more effective than neurohormonal blockade in protecting patients against a drop in EF [36].

The final class of cardio-protective treatments are modern heart failure drugs such as Entresto and SGLT2 inhibitors, but this evidence base is small and not yet ready for routine use.

The use of GLS surveillance is based on a fundamental decision to pursue selective rather than universal cardioprotection. A number of medications — angiotensin converting enzyme inhibitors, angiotensin receptor blockers, beta-adrenoceptor blockers and statins — have proven benefit in the prevention of cardiotoxicity. The use of cardioprotection in all patients at risk would be simpler than an imaging-based approach, and it avoids concerns about timing, test accuracy, and interpretation. However, the fundamental problem is that such an approach is dependent on acceptance that 80% of patients would receive this treatment unnecessarily, as cardiotoxicity develops in < 20%. In the placebo groups of randomized controlled trials, the average change of EF — while variable — is most commonly < 5% [37]. Decision analytic models have shown that a selective approach is more cost-effective than universal approach [38].

If a selective (image-guided) strategy is used, there is some time urgency in the response to an abnormal imaging surveillance test. In a study of over 200 patients with anthracycline related CTRCD, Cardinale reported 64% to respond to ACE inhibitors and beta-blockade administered within 2 months, < 30% to respond to treatment administered between 2–4 months, and < 10% to respond to treatment provided thereafter [39]. This responder status was important in predicting subsequent event-free survival, which was < 40% at 2 years in non-responders (EF < 50% and ΔEF < 10%) and partial responders (EF < 50% and Δ EF ≥ 10%).

## Conclusion

Anthracycline chemotherapy continues to carry a risk of myocardial dysfunction and heart failure. While neurohormonal antagonists and statins reduce the risk of cancer treatment-related cardiac dysfunction (CTRCD), the frequency of this problem is too low to justify the uniform use of these cardioprotective therapies. Consequently, most centers use a risk-guided strategy of cardioprotection — most commonly using 2D-EF. The problem is that 2D-EF measurements have broad confidence intervals, so substantial changes are required to designate the presence of LV dysfunction. GLS provides a more reliable and reproducible means of assessing global cardiac function, and observational studies and a randomized trial have shown the most reliable approach is to use a 10–15% relative change of GLS with therapy. GLS is a feasible, robust, and reproducible technique that requires limited training and is ready for wide adoption.
